# Profiling Clinical Research Activity at an Academic Medical Center by Using Institutional Databases: Content Analysis

**DOI:** 10.2196/12813

**Published:** 2020-08-24

**Authors:** Aisha Langford, Scott Sherman, Rachel Thornton, Kira Nightingale, Simona Kwon, Deborah Chavis-Keeling, Nathan Link, Bruce Cronstein, Judith Hochman, Howard Trachtman

**Affiliations:** 1 New York University Langone Health New York, NY United States

**Keywords:** database, clinical studies as topic, vulnerable populations, pediatrics, geriatrics

## Abstract

**Background:**

It is important to monitor the scope of clinical research of all types, to involve participants of all ages and subgroups in studies that are appropriate to their condition, and to ensure equal access and broad validity of the findings.

**Objective:**

We conducted a review of clinical research performed at New York University with the following objectives: (1) to determine the utility of institutional administrative data to characterize clinical research activity; (2) to assess the inclusion of special populations; and (3) to determine if the type, initiation, and completion of the study differed by age.

**Methods:**

Data for all studies that were institutional review board–approved between January 1, 2014, and November 2, 2016, were obtained from the research navigator system, which was launched in November 2013. One module provided details about the study protocol, and another module provided the characteristics of individual participants. Research studies were classified as observational or interventional. Descriptive statistics were used to assess the characteristics of clinical studies across the lifespan, by type, and over time.

**Results:**

A total of 22%-24% of studies included children (minimum age <18 years) and 4%-5% focused exclusively on pediatrics. Similarly, 64%-72% of studies included older patients (maximum age >65 years) but only 5%-12% focused exclusively on geriatrics. Approximately 85% of the studies included both male and female participants. Of the remaining studies, those open only to girls or women were approximately 3 times as common as those confined to boys or men. A total of 56%-58% of projects focused on nonvulnerable patients. Among the special populations studied, children (12%-15%) were the most common. Noninterventional trial types included research on human data sets (24%), observational research (22%), survey research (16%), and biospecimen research (8%). The percentage of projects designed to test an intervention in a vulnerable population increased from 17% in 2014 to 21% in 2015.

**Conclusions:**

Pediatric participants were the special population that was most often studied based on the number of registered projects that included children and adolescents. However, they were much less likely to be successfully enrolled in research studies compared with adults older than 65 years. Only 20% of the studies were interventional, and 20%-35% of participants in this category were from vulnerable populations. More studies are exclusively devoted to women’s health issues compared with men’s health issues.

## Introduction

### Background

Clinical research has spanned a wide range of activities. Projects include retrospective chart reviews, observational cohort studies, surveys and questionnaires, behavioral interventions, evaluation of educational and public service programs, investigations of normal physiology and mechanism of disease, and interventional trials of drugs and devices. The majority of these activities are conducted at academic medical centers in collaboration with other departments in the university and pharmaceutical or medical device companies.

For clinical research to truly achieve its mission of alleviating the burden of disease and improving health outcomes, it must address problems that arise throughout the population. For many years, clinical research has focused primarily on middle-aged adult men, which limited the ability to generalize to women, children, or older adults [1]. In recognition of this problem, the National Institutes of Health (NIH) required investigators to provide assurances that men and women would be eligible to participate in a planned clinical research project unless the condition being studied precluded inclusion of one gender [1,2]. Similarly, clinical research must address health problems that occur across the entire lifespan. This led to the inclusion of an additional requirement to include children in clinical research in the absence of significant risk in the pediatric age group. Finally, there are special populations that have historically been neglected and that even now are not fully included in the clinical research enterprise. Special populations are groups of individuals who may have limited access to clinical research because of physical, emotional, or socioeconomic factors that present barriers to full participation. Vulnerable groups are those that are susceptible to coercion or undue influence and have an inability to provide voluntary informed consent. Their exclusion from clinical research may be the result of barriers to participation caused by social discrimination, communication issues, language problems, lack of awareness of ongoing clinical research activity, community and cultural barriers, financial barriers (eg, inability to miss time at work and lack of back-up resources), or logistic difficulties involved in outreach to and the inclusion of these groups. Examples include older adults, immigrant groups, those with mental health disorders, and the lesbian-gay-bisexual-transgender-queer community. The relative importance is likely to vary from center to center depending upon location and the unique features of health care delivery at each site.

New York University (NYU) Langone Health serves a diverse population across the entire lifespan (Table 1).

**Table 1:** New York University Langone population statistics.

Site^a^	NYU^b^ Langone	NYU Lutheran	NYU HJD^c^	Bellevue (HHC)^d,e^	Gouverneur (HHC)^e^	Woodhull (HHC)^e^	NYU Dental	NYU Fink/Hassenfeld
Number of inpatients per year	38,000	26,500	6588	30,000	N/A^f^	14,000	N/A	N/A
Number of outpatient visits per year	912,059	620,000	227,900	492,924	250,726	385,452	392,444	14,313
Number of unique patients per year	331,034	102,067	65,972	86,961	39,372	74,495	145,532	8504
White, %	65.7	17.8	52.3	12.3	5.7	9.5	7.5	51
Black, %	8.3	18	12.6	17.2	9.1	34.1	5.5	11
Asian, %	5.1	10.1	5.5	11.5	30.4	2.6	2.7	10
Hispanic^g^, %	2.8	50	8.6	28.4	32.3	34.8	N/A	28
Native American/Pacific Islander, %	8.0	<1	0.3	N/A	N/A	N/A	<1	<1
Other or unknown, %	21.0	6	20.8	30.6	22.5	19	84.3	<1
More than 1, %	—^h^	47.4	—	—	—	—	N/A	N/A

^a^Administrative data for the period 2012-2014.

^b^NYU: New York University.

^c^HJD: Hospital for Joint Diseases.

^d^HHC: Heath+Hospitals Corporation.

^e^Includes children and adolescents.

^f^N/A: not applicable.

^g^Captured as ethnicity, not race.

^h^Data unavailable.

**Figure 1:**
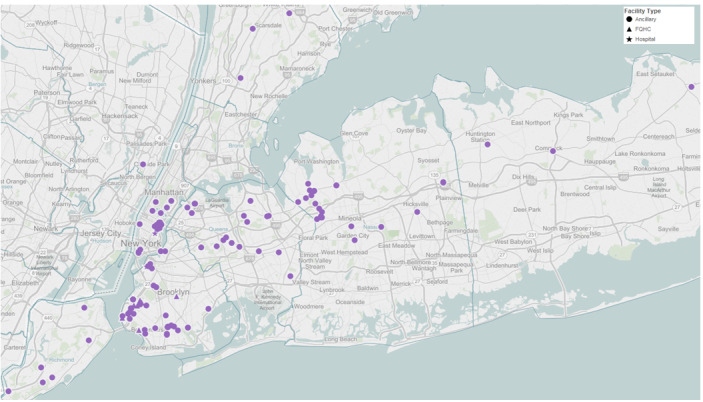
Geographical distribution of clinical sites within the New York University Langone Health network.

Moreover, the geographical distribution of sites within the NYU clinical network where care is provided and their catchment areas mirrors the diversity of the populations served (Figure 1). Although select populations such as African Americans and Asians have been studied, there has been little work at an institutional level to identify barriers and promote the inclusion of vulnerable and special populations in clinical research.

### Objectives

The New York University-Health+Hospitals (NYU-H+H) Clinical Translational Science Institute (CTSI) has been in operation for 10 years. One of the primary objectives has been to ensure that clinical research is being performed in *special or vulnerable populations* determined by the funder, which includes pediatrics and geriatrics. Therefore, we conducted the following review of clinical research performed at NYU, including the Bellevue Medical Center and Woodhull Medical Center, to provide a baseline assessment of participation by special populations. Our primary objective was to use NYU institutional administrative data to characterize the spectrum of clinical research activity and to determine whether special populations were being included in this enterprise and to enable monitoring of changes over time. A second objective was to determine if the type, initiation (enrollment of first participant), and completion (achievement of target enrollment) of studies differed by younger or older population (ie, those aged <18 years and >65 years) compared with the main population of adults aged 18 to 65 years who were not members of special populations. This profile will guide the design and implementation of programs that intend to improve participation by special populations who are underrepresented in the clinical research enterprise.

## Methods

### Data Sources

Data for this study were obtained via the NYU Langone Health’s research navigator (RNav) system. RNav was launched on November 19, 2013, and is a study management system comprising multiple modules, including institutional review board (IRB) submissions, grant proposals, a clinical research management system (CRMS), and others. All human subjects research studies that occur at NYU Langone need to be registered within RNav. In total, 2 modules were used for the collection of data for this study: (1) MyStudies for details on the study protocol and (2) CRMS, which in its current form mostly captures industry-sponsored studies and does not capture much of the clinical research conducted at NYU, was reviewed for characteristics of individual participants. MyStudies is a registration module, in which researchers summarize the study protocol as a required part of the IRB submission. CRMS is used for the capture of individual research participant information for billing compliance purposes, and therefore, it tends to be used more for clinical trials rather than population-based health studies.

A report was obtained with selected information on all studies registered in RNav as of November 2, 2016 (Multimedia Appendix 1). Data were reviewed for all studies that were IRB approved between January 1, 2014, and November 2, 2016. Studies with earlier IRB approval dates were excluded due to inconsistencies in data resulting from the transfer of data between systems upon the launch of RNav in late 2013.

### Data Collected

We collected the following demographic variables regarding participants in research: age, gender, race or ethnicity, and whether the participant was a member of a special population. The character of the research study was classified by the lead investigator of each study as observational or interventional. The latter category included studies of normal physiology, cohort studies, and population-based projects. The variables are summarized in Table 2.

As age at the time of enrollment is not a required field, an approximate age was calculated by subtracting a reference date (December 13, 2016) from the participant’s date of birth. Using this method, it is possible that the age of participants was overestimated by at most 3 years.

**Table 2:** Characteristics of clinical research captured in institutional databases.

Participants	Studies
Age	Observational Cohort Population based Data sets Survey Biospecimen Mechanistic Educational practices Health outcomes Benefit of service outcomes
Gender	Interventional Medication Device Surgical Behavioral
Race or ethnicity	N/A^a^
Member of special population	N/A

^a^N/A: not applicable.

This procedure was followed because the actual date of consent and/or enrollment was not always recorded in the system. The planned minimum and maximum age of participants, as reported by study teams, were consolidated into age groups (0-17 years, 18-39 years, 40-64 years, 65-74 years, and 75+ years). There were some pediatric studies that included participants aged under 21 years, and they were included in the first category. There were no adult studies that included patients aged under 18 years. In instances in which minimum and maximum age were obviously reversed (eg, studies with a minimum age of 90 years or maximum age of 18 years), the data were edited to correct the error. Studies with missing data were excluded from the analysis. In addition, we only reported the number of participants who enrolled into a study (ie, those who signed an informed consent) because of inconsistencies in completion of the accrual field (ie, those who were not screen failures).

To limit the analysis to those studies that had achieved the target enrollment and completed recruitment, only studies that were closed with the IRB in between 2014 and 2016 were included in the enrollment dataset.

This study was not classified as research, and the requirement for informed consent was waived by the IRB because only anonymous, aggregate data without personal health information were analyzed.

## Results

### Demographics of Patients

We examined the eligibility criteria in clinical studies that were IRB approved, whose current status was open, closed, or lapsed. Of those that were performed during the 3-year survey period of 2014-2016, 22%-24% defined the pediatric age range, 0-17 years, as the minimum age. The maximum age was 17 years in 4%- 5% of studies, which more clearly indicates the contribution of pediatric studies.

Most of the remaining studies presumably had a minimal age of 18 years because fewer than 5% specified an age above 39 years. Most of the studies included geriatric patients (age at enrollment >65 years) because the maximum projected age was ≥75 years in 64%-72% of the studies. Only 5%-12% of studies were focused exclusively on the elderly geriatric defined as minimum age above 65 years.

When examining the actual patient characteristics in studies that were closed in 2014-2016 that had individual subject data entered into CRMS, the approximate peak age at the time of enrollment was 50-59 years, with fewer participants at the pediatric and geriatric ends of the lifespan (Multimedia Appendix 2). In total, 60% of participants were aged 18-64 years at the time of enrollment, 3% were aged under 18 years, and 37% were aged 65 years or older. Initiation of recruitment (enrollment of the first participant) occurred in 31% of projects involving participants aged above 65 years versus 3% for projects involving participants aged 0-17 years. This difference occurred despite the larger number of pediatric versus geriatric studies included in the survey.

Approximately 85% of the studies were open to inclusion of both male and female participants. Of the remaining studies, those that were open only to girls or women were approximately 3 times as common as those that were confined to boys and men. Approximately 56%-58% of the projects focused on nonvulnerable patients. Among the special populations studied, children (12%-15%) were the most common. Employees, students, cognitively impaired, economically disadvantaged, and pregnant women were equally represented, 4%-7% in each subgroup, with small variations between years. Approximately one-third of the clinical studies were directed at healthy subjects and the remainder targeted individuals with specific diseases or conditions.

### Study Characteristics

Noninterventional trial types included research on human data sets (24%), observational research (22%), survey research (16%), and biospecimen research (8%). Mechanistic or physiological studies, studies involving educational practices, studies assessing expanded access or screening protocols, and those that evaluated the public benefit of service programs were infrequent.

The percentage of interventional studies among projects that did not have a vulnerable population as the primary focus was 24%-27% over the survey period. This category included drugs, devices, and surgical or behavioral interventions. This figure was higher than the percentage in projects that were designed to test an intervention in a vulnerable population. In this subgroup, the percentage of interventional trials increased from 17% in 2014 to 21% in 2015. The number ranged between 20% and 35% of all studies in most subgroups of vulnerable populations including children and cognitively impaired participants. The percentage of interventional studies was demonstrably lower (below 15%) in employees, students, and pregnant women. The number of studies performed in fetuses, neonates, and prisoners was too low to comment on the breakdown into study type.

There were no significant trends in the demographics of study participants or the type of research projects that were conducted over the 3-year study period.

This study was exempted from the requirement for ethics approval by the Institutional Review Board of NYU School of Medicine because it does not involve individual patient data.

## Discussion

### Principal Findings

This report represents a snapshot of the full gamut of clinical research activity at a large academic center. As a recipient of a Clinical Translational Science Award from the National Center for Advancing and Translational Science, NYU serves as a centralized hub capable of supporting the full spectrum of clinical investigation. Our main objectives were to characterize the spectrum of clinical research activity using institutional administrative data and to determine whether special populations were being included in this enterprise. Our main findings are as follows: (1) only 20% of the studies were interventional and 20%-35% of participants in this category of study were from special or vulnerable populations, (2) pediatric participants were the most studied special population based on the number of approved projects designed to include them, (3) fewer children than older patients were actually enrolled into approved research projects, (4) women are fully represented and more studies are exclusively devoted to women’s health issues compared with men’s health issues.

Whether administrative databases can be used to document clinical research at an institutional level may seem like a straightforward question with an obvious affirmative answer. Clinical studies are monitored from an ethical standpoint by the IRB and from a financial standpoint by grants administration offices. Registration and status reports are generally mandatory at all institutions. However, compliance is contingent upon investigator diligence and the intensity of administrative oversight. These are often less than optimal, and there can be substantial gaps in data accuracy regarding the type of study and target and actual enrollment. This is illustrated by the less than complete adherence to federal guidelines for listing clinical studies, detailing the objectives, updating enrollment, and providing final reports in a timely manner [3]. There are a number of proposals to improve the timeliness and quality of the data provided by investigators regarding their clinical research. Our findings provide an initial look at the completeness and accuracy of the data at a large academic center and provide a baseline to evaluate the efficacy of these suggestions.

The experience at NYU should have broad relevance. Historically, NYU included Bellevue Medical Center as a teaching hospital. With the recent incorporation of Lutheran Medical Center into NYU Langone Health, the patient population has become even more diverse, ethnically and economically. Thus, it is likely that issues related to clinical research identified in this report will be applicable to other institutions. Additional work is needed to determine whether the distribution of participants in clinical research matches the population served by the hospital. However, this will not detract from the availability of the full range of patient groups in the NYU-H+H CTSI.

Our inventory of clinical studies performed at NYU indicated that most of the clinical research is focused on adults and only 5% of projects are devoted to pediatric patients. Approximately 70% of studies include geriatric patients because the maximum age allowed was ≥75 years, but only 5%-12% focus exclusively on older patients (aged >65 years). This predominance of adult studies is reflected in the characteristics of the patients who were actually enrolled in the studies. Older adults were more likely to be included in the studies, but younger adults were more often the focus of studies. Thus, there was a 10-fold greater enrollment in geriatric *versus* pediatric clinical studies. Most studies included both genders, and of the remaining projects, there was a 3-fold greater number of studies that focused exclusively on women *versus* those that examined men’s health issues. There were no data regarding gender nonconforming groups. Efforts are underway to capture this information accurately without compromising participant confidentiality. Interestingly, although pediatric studies were infrequent, they represented the largest special population that was studied. This review suggests that there is a pressing need to increase the involvement of children in clinical research. The standard bias of restricting access to clinical research for participants aged under 18 years until the completion of studies in adults may need to be reconsidered. This may be especially relevant in clinical conditions in which the impact on health is as serious in children as it is in adults, such as infectious diseases [4]. This may even apply when the impact of the health problem may not be apparent in childhood, but in which the adverse consequences emerge later in life during adulthood, such as hypertension, diabetes, or obesity.

Only one-fifth of the studies performed during the survey period were interventional in nature. The representation of vulnerable populations including children and cognitively impaired individuals ranged from 20% to 35% in this category of study. This suggests that although it may be more difficult to enroll these subgroups into observational survey or biospecimens projects because of a lack of potential benefit, these individuals are being offered the opportunity and are enrolling in interventional trials. However, there are select groups such as neonates and pregnant women who may still be underrepresented in interventional clinical trials [5].

Our findings suggest that there may be a need to adopt regulatory strategies that will promote the involvement of pediatric patients in clinical research. Although our data suggest that the elderly are being included in clinical research, this claim requires ongoing reassessment as the number of patients older than 80 years continues to rise in the general population. The effect of strategies to promote the participation of underserved populations while ensuring safety and confidentiality requires real-time monitoring [6]. The advent of new integrated methods to approach patients and obtain consent for participation in clinical research, including novel uses of the electronic medical record for research (eg, direct invitations for research studies via patient portals such as Epic’s MyChart, big data mining of these clinical records), social media, and mobile devices with specific study apps, and recruitment in nonmedical centers and via direct email communication will increase the need for close surveillance to ensure efficacy and safety of all clinical research projects [7,8]. It is beyond the scope of this report to evaluate these and other novel recruitment and retention strategies, especially those that target underrepresented populations.

Approximately 30% of the trials included vulnerable populations, including children. It is unclear if this figure reflects the percentage in the general population because these individuals may be difficult to track for a variety of reasons including poor access to their place of residence, compromised mobility, and concerns raised by the individual’s legal status. The composition of this group is also likely to change over time based on the conditions that prevail generally and locally across the United States. As this group may disproportionately experience the adverse effects of common health problems, it is important to include them in clinical research activity. Potential strategies to achieve this goal include improved outreach in the less visible communities, clarification of the health problems that are key concerns, and providing legal protection to those who participate in clinical research. The efficacy of these policies needs to be evaluated systematically to ensure the selection of approaches that promote this goal.

It is important to note that the categories of vulnerable and special populations used in this report are in accordance with the objectives of the Clinical and Translational Research Unit funding opportunity guidelines, namely, the inclusion of pediatric, geriatric, and relatively inaccessible patients. This mandate provided the rationale for the formation of an Integrating Special Populations Unit in the NYU-H+H CTSI to promote recruitment of these groups. We recognize that other racial or ethnic subgroups such as African Americans and handicapped persons represent important patients who have been underrepresented in clinical research. Our study provides a benchmark for the evaluation of the participation by these other patient groups.

The gap between the initiation of clinical research studies by recruiting the first participant and completing a project by achieving the target enrollment is an important consideration. Studies that are open to enrollment for extended period of time but fail to achieve the required sample size consume valuable institutional resources. Studies like ours may provide a method to identify studies with poor recruitment, assist in designing remedial approaches to enrollment, and development of guidelines for termination of underperforming studies.

### Strengths and Limitations

There are several limitations to this study. There is a lack of detailed information about most of the studies. Moreover, there is no *gold standard* to compare information supplied by investigators with the actual number and type of clinical research being performed. However, the categories of research and the patient subgroups are generalizable, and our deidentified findings should be helpful to other institutions that are attempting to track research activity at their institution. NYU has not developed a uniform system to accurately track clinical trials, including key information about the specific target population and sample size, number of patients screened, number of patients enrolled, and number of patients studied. The CRMS in its current form mostly captures industry-sponsored studies and does not capture much of the clinical research conducted at NYU. Efforts are underway to include NIH- and foundation-sponsored projects. There is no difference in how the institution tracks studies performed in children or adults. Nonetheless, if the number of industry-sponsored projects performed varies by site or the percentage of industry-sponsored studies that are open to pediatric patients is low, these factors may impact the profile of clinical research performed at NYU versus other academic institutions in the region or more broadly around the country. In addition, we have not accounted for the number of faculty members who are involved in pediatric research compared with research on adult participants in other departments. This is an important issue, and we plan to assess this aspect of clinical research at NYU versus other institutions in a future report. The classification of studies is carried out by the lead investigator, and there may be some error in this process. During the registration of studies in the ClinicalTrials.gov registry, studies that are misclassified as interventional may be correctly listed as observational or other. However, the accuracy of other studies has not been verified. There are numerous variables being tracked, including research expenditures, IRB documentation, and participant enrollment, which are currently monitored by nonoverlapping systems. It is hoped that these important indices can be consolidated into one instrument that will improve the efficiency and accuracy of monitoring and at the same time reduce the administrative burden on the clinical research team. We are also unable to compare the clinical research activity done at NYU with the activities of other institutions in the region and across the United States. It is likely that local factors, such as the presence of competing institutions and the demographic nature of the population, influence the clinical research activity profile at any specific academic medical center. It will be important to compare the data on the scope of clinical research at single sites using institutional databases with those of mandatory clinical trial registries such as ClinicalTrials.gov [8]. We lack longitudinal data and are unable to assess the impact of the CTSI of NYU and Health+Hospitals on the volume and character of clinical research at this institution. Finally, it will be important to incorporate patients’ attitudes, including parents and other care providers, toward clinical research to gain a full perspective on the work being performed at NYU Langone Health.

We are unaware of any recent studies comparable with ours that provide a description of clinical research in a large health care system based on institutional databases. There are examples of research profiles that focus on a single disease, a defined goal, or the use of a combination of resources [9-12]. As such, this report is unique and provides a basis for comparison within our site over time and with other institutions of similar size and capacity.

### Conclusions

Using institutional databases, we documented that only 20% of the studies performed at a large, urban academic medical center were interventional and 20%-35% of participants in this category were from vulnerable populations. Although pediatric participants were the largest special population studied, they were much less likely to be included in research compared with older adults. Women are fully represented, and more studies are exclusively devoted to women’s health issues compared with men’s health issues. We anticipate that future refinements in the methodology of institutional databases will ensure that the information collected can be used to monitor research activity and guide decisions about the policies and direction of this important work. Finally, institutional databases may inform future strategies for marketing and communicating research opportunities to vulnerable populations, enhancing protocol design, and streamlining informed consent documents for clarity and understanding.

## Appendix

Multimedia Appendix 1 Template report of studies registered in Research Navigator (RNav).

Multimedia Appendix 2 Approximate age at the time of enrollment (closed studies).

## Abbreviations

CRMS: clinical research management system
CTSI: Clinical Translational Science Institute
IRB: institutional review board
NIH: National Institutes of Health
NYU: New York University
NYU-H+H: New York University-Health+Hospitals
RNav: Research Navigator

## References

[1] Geller SE, Koch A, Pellettieri B, Carnes M (2011). Inclusion, analysis, and reporting of sex and race/ethnicity in clinical trials: have we made progress?. J Womens Health (Larchmt).

[2] Nicholson LM, Schwirian PM, Groner JA (2015). Recruitment and retention strategies in clinical studies with low-income and minority populations: progress from 2004-2014. Contemp Clin Trials.

[3] Anderson ML, Chiswell K, Peterson ED, Tasneem A, Topping J, Califf RM (2015). Compliance with results reporting at ClinicalTrials.gov. N Engl J Med.

[4] Nachman S, Ahmed A, Amanullah F, Becerra MC, Botgros R, Brigden G, Browning R, Gardiner E, Hafner R, Hesseling A, How C, Jean-Philippe P, Lessem E, Makhene M, Mbelle N, Marais B, McIlleron H, McNeeley DF, Mendel C, Murray S, Navarro E, Anyalechi EG, Porcalla AR, Powell C, Powell M, Rigaud M, Rouzier V, Samson P, Schaaf HS, Shah S, Starke J, Swaminathan S, Wobudeya E, Worrell C (2015). Towards early inclusion of children in tuberculosis drugs trials: a consensus statement. Lancet Infect Dis.

[5] Cutland CL, Cunnington M, Olugbosi M, Jones SA, Hugo A, Maharaj K, Slobod K, Madhi SA (2015). Lessons learnt from enrolment and follow up of pregnant women and their infants in clinical trials in South Africa, a low-middle income country. Vaccine.

[6] Ito-Ihara T, Hong J, Kim O, Sumi E, Kim S, Tanaka S, Narita K, Hatta T, Choi E, Choi K, Miyagawa T, Minami M, Murayama T, Yokode M (2013). An international survey of physicians regarding clinical trials: a comparison between Kyoto University Hospital and Seoul National University Hospital. BMC Med Res Methodol.

[7] Frandsen M, Thow M, Ferguson SG (2016). The effectiveness of social media (Facebook) compared with more traditional advertising methods for recruiting eligible participants to health research studies: a randomized, controlled clinical trial. JMIR Res Protoc.

[8] Gupta A, Calfas KJ, Marshall SJ, Robinson TN, Rock CL, Huang JS, Epstein-Corbin M, Servetas C, Donohue MC, Norman GJ, Raab F, Merchant G, Fowler JH, Griswold WG, Fogg BJ, Patrick K (2015). Clinical trial management of participant recruitment, enrollment, engagement, and retention in the SMART study using a marketing and information technology (MARKIT) model. Contemp Clin Trials.

[9] Patel SS, Huang KE, Fleischer AB, Feldman SR (2015). Five-year trend in the number of dermatologic clinical drug trials registered on clinicaltrials.gov. J Drugs Dermatol.

[10] Cook DA, Andriole DA, Durning SJ, Roberts NK, Triola MM (2010). Longitudinal research databases in medical education: facilitating the study of educational outcomes over time and across institutions. Acad Med.

[11] Bentley SM, Melville JL, Berry BD, Katon WJ (2007). Implementing a clinical and research registry in obstetrics: overcoming the barriers. Gen Hosp Psychiatry.

[12] Witsell DL, Schulz KA, Moore K, Tucci DL, CHEER Investigators (2011). Implementation and testing of research infrastructure for practice-based research in hearing and communication disorders. Otolaryngol Head Neck Surg.

