# Supplementary material for: Profiling Clinical Research Activity at an Academic Medical Center by Using Institutional Databases: Content Analysis
Source: JMIR Public Health Surveill. 2020 Aug 24;6(3):e12813. doi: 10.2196/12813 (PMC7477669; doi:10.2196/12813)
Supplement: Multimedia Appendix 1 [file publichealth_v6i3e12813_app1.docx]

**Multimedia Appendix 1**

Template report of studies registered in Research Navigator (RNav)

| Report One (“Recruitment Report1_FromMCIT”) | | |
| --- | --- | --- |
| Field | Source | Description or comments |
| Study ID | MyStudies | N/A |
| Study title | MyStudies | N/A |
| Principal investigator | MyStudies | N/A |
| PI department | MyStudies | System-generated field based on PI selection |
| Sponsor | MyStudies | N/A |
| IRB initial approval date | IRB | N/A |
| Approval end date | IRB | N/A |
| IRB status | IRB | IRB status at the time the report was run:   - Approved; clarification requested (committee review); clarification requested (designated review); clarification requested (prereview); closed; committee review; deferred; discarded; external IRB; human research, not engaged; lapsed; modification required; modifications submitted; noncommittee review; not human research; postreview; prereview; presubmissions; terminated; withdrawn |
| CRMS status | CRMS | CRMS status at the time the report was run:   - Account setup complete awaiting finalization; changes required by Office of Clinical Trials; initial sheet; no review required; Office of Clinical Trials initial review; original version clone; reconciliation; review complete; study closed; umbrella grant; under review; withdrawn |
| Primary focus study | MyStudies | Drug, device, behavioral, surgical, or other medical intervention; mechanistic or physiologic study in humans; expanded access; biospecimen research; research on human data sets; survey or interview research; enrollment screening protocol |
| Primary disease text | MyStudies | Free text field entered by study team |
| Primary disease coded | MyStudies | Optional field for a coded primary disease |
| Gender | MyStudies | Expected gender enrollment (male, female, or both) |
| Maximum age | MyStudies | Driven by I/E criteria |
| Minimum age | MyStudies | Driven by I/E criteria |
| Healthy volunteers’ study | MyStudies | Will healthy volunteers be enrolled (yes or no) |
| Vulnerable population | MyStudies | Vulnerable populations targeted by the study (no vulnerable populations; children; cognitively impaired; economically disadvantaged; employees; fetuses; neonates; pregnant women; prisoners; students) |
| Projected subjects to sign consent | MyStudies | Projected number of subjects to sign the consent form |
| Projected accrual (NYU) | MyStudies | Projected number of subjects to be accrued at NYU |
| Projected accrual (All sites) | MyStudies | Projected number of subjects to be accrued at all sites |
| Report Two (“Recruitment Report2_FromMCIT”) | | |
| Field | Source | Description or comments |
| Study ID | MyStudies | N/A |
| Trial ID | CRMS | CRMS-specific ID (ex: S14-0001 is c14-0001) |
| PI name | MyStudies | N/A |
| PI department | MyStudies | N/A |
| Sponsor | Not known | This field exists in MyStudies, IRB, and CRMS and is not synchronized. Not sure which module sponsor was pulled from in this report—we can specify in subsequent reports |
| IRB initial approval date | IRB | N/A |
| Approval end date | IRB | N/A |
| IRB status | IRB | IRB status at the time the report was run:   - Approved; clarification requested (committee review); clarification requested (designated review); clarification requested (prereview); closed; committee review; deferred; discarded; external IRB; human research, not engaged; lapsed; modification required; modifications submitted; noncommittee review; not human research; postreview; prereview; presubmissions; terminated; withdrawn |
| Subject ID | CRMS | N/A |
| Date of birth | CRMS | Date of birth for each subject entered into CRMS |
| Ethnicity | CRMS | Ethnicity for each subject entered into CRMS |
| Race | CRMS | Race for each subject entered into CRMS |
| Registration type | CRMS | Standard (allows study team to enter in demographic and individual visit information); minimal (subject ID and enrollment date only); tally (just a single number that is updated periodically to reflect current number of subjects enrolled) |
| Actual number of subjects to sign consent | CRMS | As entered into CRMS |
| Actual accrual (NYU) | CRMS | As entered into CRMS |
| Actual accrual (all sites) | CRMS | As entered into CRMS |
