# Supplementary figures and images for: Profiling Clinical Research Activity at an Academic Medical Center by Using Institutional Databases: Content Analysis
Source: JMIR Public Health Surveill. 2020 Aug 24;6(3):e12813. doi: 10.2196/12813 (PMC7477669; doi:10.2196/12813)

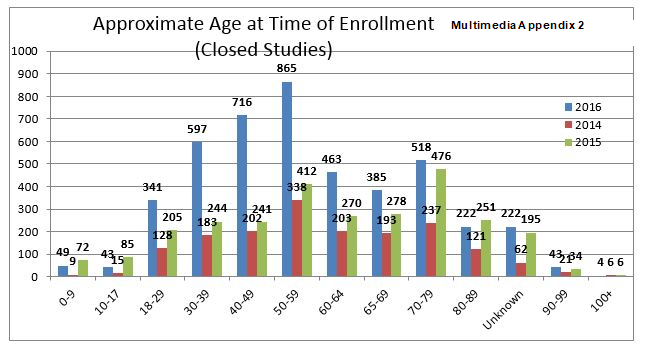

Supplement: Multimedia Appendix 2 [file publichealth_v6i3e12813_app2.png]
